# Identification of amino acid residues in the MT-loop of MT1-MMP critical for its ability to cleave low-density lipoprotein receptor

**DOI:** 10.3389/fcvm.2022.917238

**Published:** 2022-08-25

**Authors:** Maggie Wang, Adekunle Alabi, Hong-mei Gu, Govind Gill, Ziyang Zhang, Suha Jarad, Xiao-dan Xia, Yishi Shen, Gui-qing Wang, Da-wei Zhang

**Affiliations:** ^1^The Department of Pediatrics and Group on the Molecular Cell Biology of Lipids, Faculty of Medicine Dentistry, College of Health Sciences, University of Alberta, Edmonton, AB, Canada; ^2^Department of Orthopedics, The Sixth Affiliated Hospital of Guangzhou Medical University, Qingyuan People’s Hospital, Qingyuan, China

**Keywords:** LDL clearance, lipid metabolism, atherosclerosis, receptor shedding, metalloproteinase, alanine scanning, lipoprotein metabolism

## Abstract

Low-density lipoprotein receptor (LDLR) mediates clearance of plasma LDL cholesterol, preventing the development of atherosclerosis. We previously demonstrated that membrane type 1-matrix metalloproteinase (MT1-MMP) cleaves LDLR and exacerbates the development of atherosclerosis. Here, we investigated determinants in LDLR and MT1-MMP that were critical for MT1-MMP-induced LDLR cleavage. We observed that deletion of various functional domains in LDLR or removal of each of the five predicted cleavage sites of MT1-MMP on LDLR did not affect MT1-MMP-induced cleavage of the receptor. Removal of the hemopexin domain or the C-terminal cytoplasmic tail of MT1-MMP also did not impair its ability to cleave LDLR. On the other hand, mutant MT1-MMP, in which the catalytic domain or the MT-loop was deleted, could not cleave LDLR. Further Ala-scanning analysis revealed an important role for Ile at position 167 of the MT-loop in MT1-MMP’s action on LDLR. Replacement of Ile167 with Ala, Thr, Glu, or Lys resulted in a marked loss of the ability to cleave LDLR, whereas mutation of Ile167 to a non-polar amino acid residue, including Leu, Val, Met, and Phe, had no effect. Therefore, our studies indicate that MT1-MMP does not require a specific cleavage site on LDLR. In contrast, an amino acid residue with a hydrophobic side chain at position 167 in the MT-loop is critical for MT1-MMP-induced LDLR cleavage.

## Introduction

Plasma levels of low-density lipoprotein cholesterol (LDL-C) are positively correlated to the risk of cardiovascular disease. Hepatic LDL receptor (LDLR) is the main pathway that clears plasma LDL. Mutations in LDLR cause familial hypercholesterolemia (FH), characterized by elevated levels of plasma LDL-C and increased risk of coronary heart disease (1). Structurally, LDLR’s ectodomain consists of 7 ligand-binding repeats (LR) at its N terminus region for binding substrates, such as interacting with apolipoprotein (apo) B100 on LDL and apoE on lipoprotein remnants. LR is followed by the epidermal growth factor precursor homology (EGF) domain that is required for lipoprotein release in the endosome and recycling of LDLR. Upon substrate binding, LDLR is endocytosed into a clathrin-coated vesicle and then delivered to the endosome. In the acidic environment of endosomes, LDL is released from the receptor, and LDLR is recycled back to the cell surface. LDL is then shuttled to the lysosome for degradation. Further down in the primary sequence is the clustered O-linked glycosylation region, whose function is still elusive, followed by the transmembrane domain and a cytoplasmic tail at the C terminus. The NPVY motif within the C-terminus is critical for the internalization of the receptor into clathrin-coated pits (2–4). Substitution of Tyr807 to cysteine, otherwise known as the J.D. mutation in FH, impairs LDLR endocytosis but does not affect LDLR trafficking to the cell surface or the binding of LDL to the receptor (3).

Currently, the primary lipid-lowering therapy is statins, which inhibit 3-hydroxy-3-methyl-glutaryl-CoA reductase (HMGCR), the rate-limiting enzyme in cholesterol biosynthesis. Reduction in cellular cholesterol levels activates the transcriptional activity of sterol regulatory element-binding protein 2 (SREBP2), thereby increasing LDLR expression and subsequent LDL-C clearance (1, 5, 6). However, the efficiency of statin treatment is approximately 20–40%. Furthermore, about 15% of people treated with statins show intolerance to treatment and require alternative therapies to lower LDL-C (7). One option is to target proprotein convertase subtilisin/kexin type 9 (PCSK9), which promotes LDLR degradation (8–12). While recently approved PCSK9 inhibitors can effectively reduce plasma LDL-C levels (13, 14), the treatment is expensive. PCSK9 siRNA therapy may be more affordable, but it is still too financially unsustainable for all eligible patients to use in primary prevention. Therefore, the search for alternative treatments is necessary.

The ectodomain of LDLR can be cleaved by proteases to generate a soluble form of LDLR (sLDLR), which inactivates LDLR-mediated LDL clearance. It has been reported that plasma levels of sLDLR are positively correlated to circulating LDL-C levels (15–17). In our previous studies, we found that membrane type 1-matrix metalloproteinase (MT1-MMP) promotes ectodomain shedding of LDLR, thereby increasing plasma LDL-C levels and exacerbating the development of atherosclerosis in mice (18, 19). Therefore, targeting MT1-MMP has the potential to be a new lipid-lowering strategy.

MT1-MMP belongs to the MMP family that consists of 23 zinc-dependent endopeptidases in humans. It plays essential physiological roles in tissue remodeling and development by cleaving extracellular matrix components and non-matrix substrates (20–22). Structurally, MT1-MMP contains an N-terminal signal peptide, a prodomain, and then a catalytic domain with the conserved zinc-binding site (HE240XGHXXGXXH). Next is a flexible hinge region, a hemopexin (HPX) domain that links the catalytic domain, a transmembrane domain that anchors the protein to cell membranes, and a C-terminal cytoplasmic tail involved in endocytosis of MT1-MMP (19, 23–25). The catalytic domain is highly conserved among MMP family members (19, 24). Clinical trials of all broad-spectrum MMP inhibitors that target the catalytic domain in oncology have failed due to lack of inhibitor specificity (26). On the other hand, exosites that are regions outside the catalytic core domain of MMPs are less conserved and contribute to substrate selection and binding (27–29). Therefore, drugs targeting exosites have a great potential to be both MMP- and substrate-specific.

In this study, we investigated the regions in LDLR and MT1-MMP required for MT1-MMP-induced LDLR cleavage. We found that deletion of the MT-loop (amino acids 163-170) within the catalytic domain of MT1-MMP impaired its ability to cleave LDLR. Alanine scanning revealed that Ile at position 167 within the MT-loop played an important role in MT1-MMP-promoted LDLR degradation. Given that the MT-loop is specific for MT1-MMP, our findings provide a foundation for the future design of inhibitors that can specifically target MT1-MMP’s action on LDLR.

## Materials and methods

### Materials

Cell culture medium, fetal bovine serum (FBS), Lipofectamine 3000, GeneJet and PureLink™ Hipure plasmid miniprep kit were obtained from ThermoFisher Scientific. Complete EDTA-free protease inhibitors were purchased from Roche. All other reagents were obtained from Fisher Scientific unless otherwise indicated.

The following antibodies were used: HL-1, a mouse monoclonal anti-the linker sequence between ligand binding repeat (LR) 4 and LR5 of LDLR antibody (30, 31); a rabbit anti-MT1-MMP monoclonal antibody (Abcam, ab51074); a mouse anti-MT1-MMP monoclonal antibody (EMD Millipore, MAB3329); a rabbit anti-HA polyclonal antibody (ProteinTech, 51064-2-AP); a mouse anti-HA monoclonal antibody (ProteinTech, 66006-2-lg); a Dylight™ 680-conjugated rabbit anti-HA antibody (Rockland, 600-444-384); a Dylight™ 800-conjugated rabbit anti-HA antibody (Rockland, 600-445-384); a mouse anti-actin monoclonal antibody (ProteinTech, 66009-1-Ig); a mouse anti-Na + /K + -ATPase antibody (BD Biosciences, 610993); and a mouse anti-transferrin receptor monoclonal antibody (BD Biosciences, 612125).

### Site-directed mutagenesis

Plasmid containing cDNA of the full-length human MT1-MMP (NM_004995) with an HA-tag between Asp115 and Glu116 (a kind gift from Dr. Weiss, University of Michigan) was used as the template to generate the mutant form of MT1-MMP. Plasmid pBudCE4.1 containing cDNA of the full-length human LDLR (NM_000527) with an N-terminal HA-tag was used to create the mutant forms of LDLR. Mutagenesis was performed using QuickChange Lightning site-directed mutagenesis kit (Agilent Technologies) as described in our previous studies (32–34). LDLR deletions were generated as described previously (31, 35). To make the catalytic deletion of MT1-MMP (ΔCAT), an *Age*I site was introduced at Tyr112 and Pro312, respectively. To delete the hemopexin-like repeats in MT1-MMP (ΔHPX), an *Age*I site was introduced at Pro312 and Gly535, respectively. The resulting construct was digested with *Age*I (FastDigest *Bsh*TI, Thermo Scientific) and ligated using the Quick Ligation Kit (New England Biolabs). To remove the C-terminal cytoplasmic region of MT1-MMP, a stop codon was introduced at Arg563. The sequences of the oligonucleotides containing the residues to be mutated were synthesized by IDT (Coralville, IA) and listed in Table 1. The presence of the desired mutation and the integrity of each construct were verified by DNA sequencing. cDNA of human MT1-MMP and human LDLR is consistent with DNA sequence in NCBI database, human MT1-MMP: NM_004995; human LDLR: NM_000527.

**TABLE 1:** Primers.

MT1-MMP
E240A: 5′- GGT GGC TGT GCA CGC GCT GGG CCA TGC CC -3′; Y112-*Age*I: 5′-GTT CGA AGG AAG CGC ACC GGT ATC CAG GGT CTC-3′; P312-*Age*I: 5′-GAT AAA CCC AAA AAC ACC GGT TAT GGG CCC AAC-3′; Gly535-*Age*I: 5′-GAC GAG GAG GGC ACC GGT GCG GTG AGC GCG G-3′; Arg563-Stop: 5′-CAG TCT TCT TCT TCT GAC GCC ATG GGA C -3′; MT-loop deletion: 5′- CCA CTG CGC TTC CGC GAG GTG CAT GAG AAG CAG GCC GAC ATC ATG ATC -3′; Pro163: 5′-CTT CCG CGA GGT GGC CTA TGC CTA CAT C-3′; Y164-A: 5′-CCG CGA GGT GCC CGC TGC CTA CAT CCG TG-3′; Y166-A: 5′-GAG GTG CCC TAT GCC GCC ATC CGT GAG GGC-3′; I167-A: 5′-GTG CCC TAT GCC TAC GCC CGT GAG GGC CAT G-3′; R168-A: 5′-CCC TAT GCC TAC ATC GCT GAG GGC CAT GAG-3′; E169-A: 5′-CTA TGC CTA CAT CCG TGC GGG CCA TGA GAA G-3′; G170-A: 5′-GCC TAC ATC CGT GAG GCC CAT GAG AAG CAG-3′; I167-L: 5′-GTG CCC TAT GCC TAC CTC CGT GAG GGC CAT G-3′; I167-V: 5′-GTG CCC TAT GCC TAC GTC CGT GAG GGC CAT G-3′; I167-M: 5′-GTG CCC TAT GCC TAC ATG CGT GAG GGC CAT G-3′; I167-F: 5′-GTG CCC TAT GCC TAC TTC CGT GAG GGC CAT G-3′; I167-E: 5′-GTG CCC TAT GCC TAC GAA CGT GAG GGC CAT GAG-3′; I167-K: 5′-GTG CCC TAT GCC TAC AAG CGT GAG GGC CAT GAG-3′; I167-T: 5′-GTG CCC TAT GCC TAC ACC CGT GAG GGC CAT G-3′; V162-A: 5′-G CGC TTC CGC GAG GCG CCC TAT GCC TAC ATC-3′
**LDLR**
A521V: 5′-CTC CAA GCC AAG GGT CAT CGT GGT GGA T-3′; G529V: Forward-5′-GTG GAT CCT GTT CAT GTC TTC ATG TAC TGG-3′; I566AT567A: CAG TGG CCC AAT GGC GCC GCC CTA GAT CTC CTC AGT; N645V: 5′-AAC TTG TTG GCT GAA GTC CTA CTG TCC CCA-3′; A789V: 5′-CAG TAG CGT GAG GGT TCT GTC CAT TGT C-3′

### Cell culture, transfection, and immunoblotting

HEK293 cells were maintained in DMEM (high glucose) containing 10% (v/v) FBS at 37°C in a 5% CO_2_ humidified incubator. Plasmid DNA was introduced into cells using polyethylenimine (PEI, Mw 25000; DNA:PEI = 1:3) or Lipofectamine™ 3000 as described (36). 48 h after treatment, whole cell lysate was prepared using a lysis buffer (1% Triton X-100, 150 mM NaCl, 50 mM Tris, pH 7.4) containing 1 × Complete EDTA-free protease inhibitors as described in our previous studies (37). Protein concentrations were determined by the BCA protein assay. Equal amount of whole cell lysate was subjected to SDS-PAGE and transferred to nitrocellulose membranes by electroblotting. Immunoblotting was performed using antibodies as indicated. Antibody binding was detected using IRDye-labeled goat anti-mouse or anti-rabbit IgG antibody (Li-Cor). The signals were detected and quantified on a Li-Cor Odyssey Infrared Imaging System (Li-Cor).

### Biotinylation

HEK293 cells in 6-well plates were transfected with plasmids containing cDNAs for wild type or mutant LDLR and/or wild type or mutant MT1-MMP using Lipofectamine 3000. After 48 h, cell surface proteins were biotinylated with Sulfo-NHS-LC-Biotin for 20 min at room temperature exactly as described (33, 37). After quenching in 1 x PBS containing 100 mM glycine and washing in 1 x PBS, the cells were lysed in 200 μl of lysis buffer and then subjected to centrifugation at 15,000 rpm for 5 min. The supernatant was incubated with Neutravidin agarose (Pierce, 30 μl of 50% slurry). The mixture was rotated overnight at 4°C. After washing in lysis buffer three times, the cell surface proteins were eluted from the beads and then analyzed by SDS-PAGE and immunoblotting.

### Migration assay

The experiment was carried out using the transwell migration assay (38). HEK293 cells were seeded on a 6-well cell culture plate, and then transfected with empty plasmid or plasmid containing the wild-type or mutant MT1-MMP using Lipofectamine™ 3000. 24 h later, cells were trypsinized and counted. Equal numbers of cells in 500 μl of serum-free DMEM medium were placed on a cell culture insert pre-coated with collagen type I (8 μm). 500 μl of DMEM containing 20% FBS was placed below the insert. 48 h after, the insert was rinsed briefly in 1X PBS, then fixed and stained with crystal violet in 20% methanol. Cells on the top of the insert were removed with a cotton swab. Cells on the bottom of the insert were imaged on an OMAX M837ZL-C140U3 microscope and counted (10 images per insert). Relative cell numbers were calculated by dividing the average cell numbers by the image area that was measured with OMAX ToupView.

### Immunofluorescence

Confocal microscopy was carried out as described previously (37, 39, 40). Briefly, HEK293 cells cultured on a coverslip were transfected with empty vector or plasmid containing the wild-type or mutant HA-MT1-MMP cDNA. 48 h later, cells were fixed with 3% paraformaldehyde in PBS, and then permeabilized using ice-cold methanol. After blocking, the cells were incubated with an anti-HA polyclonal antibody (1:100) and an anti-Na^+^/K^+^-ATPase monoclonal antibody (1:100). Antibody binding was detected using Alexa Fluor 488 goat anti-rabbit IgG and Alexa Fluor 568 goat anti-mouse IgG. Nuclei were stained with 4′, 6-diamidino-2-phenylindole (DAPI, ThermoFisher). Coverslips were mounted on the slides with ProLong Diamond Antifade Mountant (ThermoFisher). Localization of MT1-MMP in the transfected cells was determined using a Leica SP5 laser scanning confocal microscope (filters: 405 nm for DAPI, 488 nm for Fluor 488, 594 nm for Fluor 568).

### Statistical analysis

Statistical analyses were performed using GraphPad Prism version 9.0 (GraphPad Software). The significant difference between two groups was determined *via* Student’s *t*-test and defined as *p < 0.05, ^**^p < 0.01, ^***^p < 0.001, and ^****^p < 0.0001. *p* > 0.05 was defined as no significance. Values of all data, unless otherwise indicated, were depicted as mean ± S.D. All experiments, unless indicated, were repeated at least three times.

## Results

### Specific sites on low-density lipoprotein receptor for MT1-MMP-induced cleavage

**FIGURE 1:**
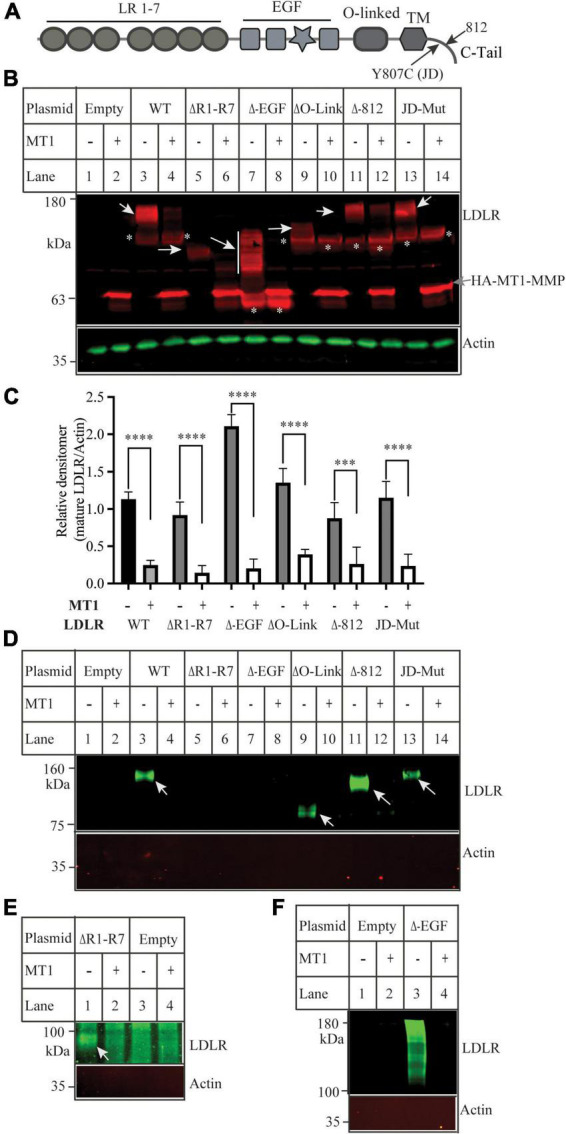
Effects of mutations in low-density lipoprotein receptor (LDLR) on MT1-MMP-induced LDLR cleavage **(A)** Diagram of the functional domains of LDLR. LR, ligand binding region. EGF, the epidermal growth factor precursor homology domain. O-linked, the O-linked sugar domain. TM, transmembrane domain. JD, the J.D. mutation (Y807C) identified in a FH patient. C-tail, the C-terminal cytoplasmic tail. **(B**) Immunoblotting and **(C)** quantification. Equal amount of plasmid DNA containing the wild-type (WT) or mutant LDLR deletion cDNA and empty (-) or plasmid containing the wild-type MT1-MMP (MT1) were transfected into HEK293 cells using PEI. 48 h later, whole cell lysate was prepared, and equal amount of total proteins in whole cell lysates was applied to SDS-PAGE and immunoblotting with antibodies as indicated. HA-tagged MT1-MMP and LDLR were detected by a Dylight 680-conjugated rabbit anti-HA antibody (Rockland, 600-444-384) and 3143, respectively. The mature and premature forms of the wild type and mutant LDLR were indicated by white arrows and *, respectively. MT1-MMP was indicated by a gray arrow. Actin was detected by a mouse anti-actin monoclonal antibody. **(D–F)** Biotinylation. HEK293 cells were co-transfected with the WT or mutant LDLR and empty (-) or WT MT1-MMP (MT1) using Lipofectamine 3000. 48 h after, the cells were subjected to biotinylation. Samples were heated at 85 **(D,E)** or 37°C **(F)** to elude proteins from Neutravidin beads and then subjected to immunoblotting using a mouse anti-LDLR (HL-1) **(D,F)**, or a rabbit anti EGFA of LDLR **(E)**. Actin was detected with a mouse anti-actin antibody. Similar results were observed in three different experiments. Representative images were shown **(B,D–F)**. Densitometry was determined by a Li-Cor Odyssey Infrared Imaging System. Relative densitometry was defined as the ratio of the densitometry of the mature form of wild-type or mutant LDLR to that of Actin at the same condition **(C)**. Values were mean ± S.D. of ≥3 experiments. Significance was defined as the mature form of LDLR in the presence of MT1-MMP vs. the mature form of LDLR in the absence of MT1-MMP. ***p < 0.001, ****p < 0.0001.

We have previously reported that MT1-MMP mediates shedding of LDLR and regulates plasma LDL-C metabolism (18). Here, we sought to identify the specific sites on LDLR for MT1-MMP-induced cleavage. LDLR has five distinct regions, each playing different critical roles in the functionality of the protein. To determine if any of these regions were important for MT1-MMP-mediated LDLR shedding, we made various LDLR mutants with deletions of different LDLR function domains, including the ligand binding repeat deletion (ΔR1-R7), EGF-like domain deletion (ΔEGF), O-Linked glycosylation domain (ΔO-Link), and C-terminal domain (Δ812) (Figure 1A). In addition, to test if endocytosis of LDLR plays a role in MT1-MMP-mediated LDLR cleavage, we generated the J.D. mutant LDLR, where the Tyr in the NPVY motif is mutated to Cys (Y807C). The J.D. mutant (JD-Mut) is a naturally occurring mutation that impairs LDLR endocytosis and consequently results in FH. However, the mutation does not affect trafficking of LDLR to plasma membrane (3). Each mutation was co-transfected with an equal amount of empty or the wild-type MT1-MMP-containing plasmid into HEK293 cells using PEI. MT1-MMP has a HA-tag in the HPX region (41). MT1-MMP and LDLR were detected by a rabbit anti-HA polyclonal antibody and a rabbit anti-LDLR antibody, 3143 that recognizes the C-terminal region of LDLR (31, 42), respectively. A IRDye-680 labeled goat anti-rabbit IgG recognizes both LDLR and MT1-MMP. LDLR is a glycosylated protein, and glycosylation starts in the endoplasmic reticulum (ER) and is matured in the Golgi. The wild-type and mutant LDLR, except for the ΔR1-R7 mutant, displayed well-separated premature (p) and mature (m) forms; top one was the fully glycosylated mature form (indicated by white arrows), while the bottom band was the under glycosylated premature form (indicated by *). We did not observe the premature form of the ΔR1-R7 mutant (lanes 5 and 6), which is consistent with our previous study (35). The mature form of the ΔEGF mutant showed a smeared pattern possibly due to altered glycosylation (lane 7), while the mature form of the ΔO-Link mutant was weak and very close to its premature form since LDLR is primarily O-glycosylated at the side chain of Ser and Thr residues in the O-Linked glycosylation domain (43, 44). Nevertheless, the mature form of the wild-type LDLR, all deletion mutants, and the JD mutant was significantly reduced by MT1-MMP (Figures 1B,C). We also performed a biotinylation experiment to monitor cell surface proteins. LDLR was detected by a mouse monoclonal antibody, HL-1 that recognizes the linker between ligand-binding repeat 4 and 5. We could detect LDLR but not actin in the biotinylation experiment (Figure 1D), indicating biotinylation of cell surface proteins. We observed that the cell surface wild-type and mutant LDLR, including ΔO-Link, Δ812 and JD mutation, were markedly reduced in cells expressing MT1-MMP (Figures 1D). ΔR1-R7 cannot be recognized by HL-1. When the membrane was blotted with a rabbit anti-EGFA of LDLR antibody, we found that MT1-MMP promoted degradation of cell surface ΔR1-R7 [Figure 1E, lane 2 vs. 1)]. On the other hand, we observed that the majority of biotinylated ΔEGF, unlike ΔEGF in whole cell lysate (Figure 1B), was aggregated with a small portion of smeared protein bands above the 100 kDa marker even though the samples were heated at 37°C for 5 min (Figure 1F, lane 3), while no heating could not elude biotinylated ΔEGF from NeutrAvidin beads (data not shown). However, all these signals were markedly reduced in cells expressing MT1-MMP (Figure 1F, lane 4 vs. 3), indicating that MT1-MMP reduced cell surface ΔEGF. Therefore, each of these regions and endocytosis of LDLR are not required for MT1-MMP’s action on the receptor.

**FIGURE 2:**
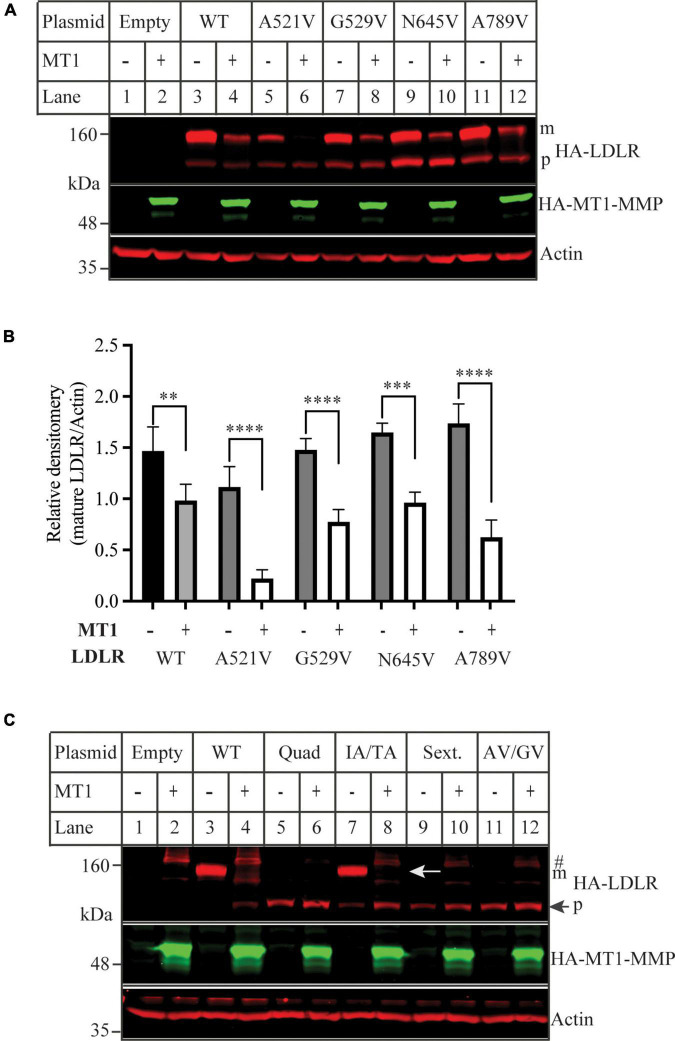
Mutations of predicted MT1-MMP cleavage sites on LDLR **(A,C)** immunoblotting and **(B)** quantification. HEK293 cells were co-transfected with an equal amount of the wild-type (WT) or mutant LDLR and empty (-) or the wild type MT1-MMP (MT1) plasmid using PEI. 48 h later, whole cell lysate was isolated and applied to immunoblotting. After transfer, the membrane was cut into halves above the 75 kDa protein marker. HA-tagged LDLR on the top membrane was detected with a Dylight™ 680-conjugated rabbit anti-HA antibody (Rockland, 600-444-384). m: mature form of LDLR; p: premature form of LDLR. MT1-MMP on the bottom was detected by a Dylight™ 800-conjugated rabbit anti-HA antibody (Rockland, 600-445-384). Similar results were observed in three different experiments. Representative images were shown in **(A,C)**. Densitometry was determined by a Li-Cor Odyssey Infrared Imaging System. Relative densitometry was defined as the ratio of the densitometry of the mature form of wild-type or mutant LDLR to that of Actin at the same condition **(B)**. Values were mean ± S.D. of ≥3 experiments. Significance was defined as the mature form of LDLR in the presence of MT1-MMP vs. the mature form of LDLR in the absence of MT1-MMP. **p < 0.01, ***p < 0.001, ****p < 0.0001.

We then used the software CleavePredict to predict the cleavage sites of MT1-MMP on LDLR. CleavePredict is a validated free access web server for predicting the substrate cleavage pattern by matrix metalloproteinases (MMP). It employs MMP specific position weight matrices, which is derived from statistical analysis of high-throughput phage display experimental cleavage results of metalloproteinases (45). The software predicts 22 putative MT1-MMP cleavage sites on LDLR, with a spread across all its extracellular domain (Table 2). Based on the position weight matrix score and proximity to the transmembrane domain, which has been suggested to be the cleavage region of MT1-MMP on LDL receptor related protein 1 (LRP1) (46), we selected 5 locations A521, G529, G565, and N645 within the YWTD region and A789 within the transmembrane domain near the outer layer of plasma membrane. The key residue at positions 521, 529, 645, and 789 was individually replaced with Val and I566 and T567 were replaced with Ala to disrupt each of the putative cleavage sites as confirmed by CleavePredict. We then co-expressed the mutant LDLR with the wild-type MT1-MMP in HEK293 cells. As shown in Figures 2A,B, the mature form of all mutant LDLR tested was effectively reduced by MT1-MMP. MT1-MMP also reduced the levels of the mature form of mutant I556AT567A (Figure 2C, lane 8 vs. 7). To determine whether the loss of one predicted cleavage site was compensated by the remaining predicted cleavage sites, we made a sextuple (Sext.) mutation to remove all the five predicted sites (A521V, G529V, I556A, T567A, G565, and N645), a quadruple (Quad.) mutation (A521V, G529V, G565, and N645), and a double mutation A521V/G529V (AV/GV). We observed the premature but not the mature form of all these mutations, and, as expected, the premature form was not affected by MT1-MMP (Figure 2C, lanes 6 vs. 5, 10 vs. 9, and 12 vs. 11). These observations indicate that these mutations appeared to impair LDLR trafficking to the plasma membrane. Given that MT1-MMP promotes cleavage of the mature form of LDLR, the impact of these mutations on MT1-MMP-induced LDLR cleavage cannot be determined. Therefore, it is possible that none or more than one of these predicted sites are involved in MT1-MMP-induced LDLR cleavage. The loss of each of the predicted cleavage sites may be compensated by the remaining predicted sites. Nevertheless, our findings suggest that none of the five predicted cleavage points individually is necessary for MT1-MMP-induced LDLR cleavage.

**TABLE 2:** Predicted MT1-MMP cleavage site on LDLR as determined by the online CleavePredict software, showing cleavage positions, residues and position weight matrix score.

P1 cleavage positions	Residues	PWM^Score
14	WTVAL-LLAAA	0.54
86	CIPQF-WRCDG	1.94
152	CGPAS-FQCNS	5.51
186	QRCRG-LYVFQ	2.87
397	KAVGS-IAYLF	1.14
400	GSIAY-LFFTN	3.05
421	SEYTS-LIPNL	2.97
425	SLIPN-LRNVV	2.30
**521**	**SKPRA-IVVDP**	**4.90**
**529**	**DPVHG-FMYWT**	**5.37**
541	GTPAK-IKKGG	1.95
554	VDIYS-LVTEN	1.17
**565**	**QWPNG-ITLDL**	**5.36**
584	SKLHS-ISSID	3.26
608	RLAHP-FSLAV	2.38
610	AHPFS-LAVFE	1.45
**645**	**LLAEN-LLSPE**	**5.96**
657	VLFHN-LTQPR	4.07
685	CLPAP-QINPH	2.54
701	ACPDG-MLLAR	0.81
707	LLARD-MRSCL	1.93
**789**	**SSVRA-LSIVL**	**7.55**

Selected positions were highlighted in bold.

### The requirement of domains in MT1-MMP for low-density lipoprotein receptor shedding

**FIGURE 3:**
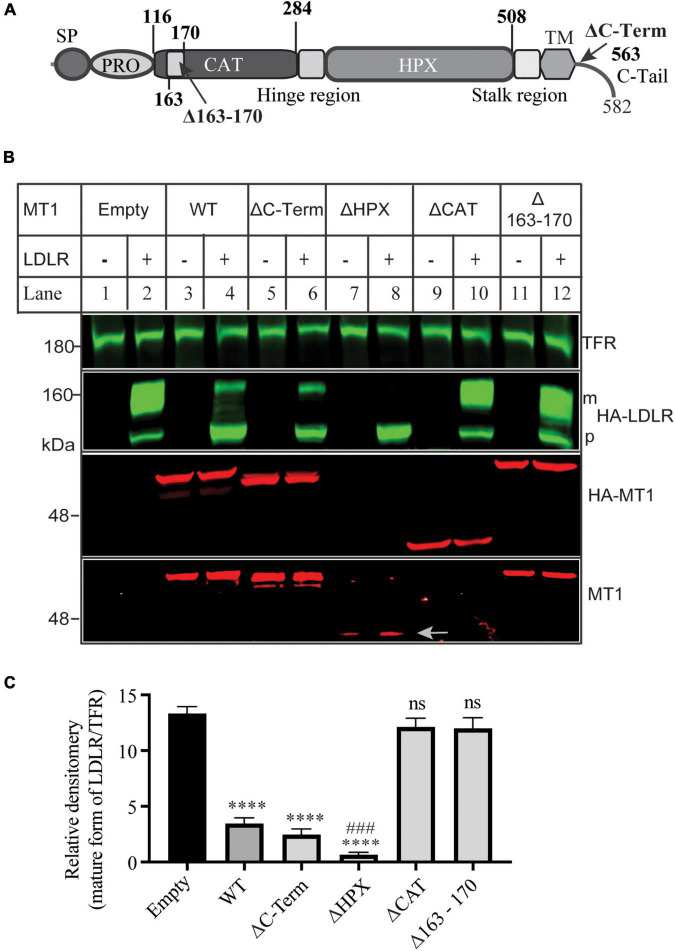
Effects of mutations in MT1-MMP on its ability to cleave LDLR **(A)** Diagram of the functional domains of MT1-MMP. SP, signal peptide. PRO, prodomain. CAT, the catalytic domain. HPX, the hemopexin domain. TM, transmembrane domain. C-Tail, the C-terminal cytoplasmic tail. **(B**) Immunoblotting and **(C)** quantification. Equal amount of plasmid DNA containing the wild-type or mutant MT1-MMP and empty plasmid (-) or the wild-type LDLR-containing plasmid were co-transfected into HEK293 cells using PEI. 48 h later, whole cell lysate was prepared, and equal amount of total proteins in whole cell lysate was applied to immunoblotting. After transfer, the membrane was cut into halves above the 75 kDa protein marker. The top membrane was blotted with a Dylight™ 800-conjugated rabbit anti-HA antibody to recognize HA-LDLR. m: mature form of LDLR; p: premature form of LDLR. The bottom membrane was blotted with a Dylight™ 680-conjugated rabbit anti-HA antibody to detect HA-MT1-MMP (HA-MT1). The bottom part was also blotted with a mouse anti-MT1-MMP antibody to detect MT1-MMP (MT1) (EMD Millipore, MAB3329). TFR, transferrin receptor (TFR). Representative images were shown **(B)**. Densitometry was determined by a Li-Cor Odyssey Infrared Imaging System. Relative densitometry was defined as the ratio of the densitometry of the mature form (m) of LDLR to that of TFR at the same condition **(C)**. Values were mean ± S.D. of ≥3 experiments. Significance was defined as the mature form of LDLR in the presence of the wild-type or mutant MT1-MMP vs. the mature form of LDLR in the absence of MT1-MMP (Empty). ns (vs. Empty), no significance, *p* > 0.05. **** (vs. Empty), *p* < 0.0001. ### [ΔHPX vs. wild type MT1-MMP (WT)], *p* < 0.001.

Next, we determined which functional domains in MT1-MMP were critical for its ability to cleave LDLR. Thus, we deleted the C-terminal cytoplasmic region (ΔC-Term), the hemopexin-like repeats (ΔHPX), the catalytic domain (ΔCAT), and the MT-loop within the catalytic domain (Δ163-170) (Figure 3A). The wild-type or mutant MT1-MMP was co-expressed with the wild-type LDLR in HEK293 cells. The expression of the wild-type and mutant MT1-MMP except for ΔHPX that did not have the HA-tag, was confirmed by an anti-HA antibody, while a mouse anti-MT1-MMP monoclonal antibody that recognizes the catalytic domain revealed the wild-type, ΔC-Term, ΔHPX, and Δ163-170 but not ΔCAT MT1-MMP (Figure 3B). We found that removal of the C-terminal cytoplasmic region (ΔC-Term) and the hemopexin-like repeats (ΔHPX) did not significantly impair MT1-MMP-induced reduction in the mature form of LDLR (Figures 3B,C). Interestingly, ΔHPX appeared to reduce the abundance of the mature form of LDLR more significantly than the wild-type MT1-MMP (lane 8 vs. 4) even though its expression was lower than the wild-type protein (indicated by an arrow in lanes 7 and 8). On the other hand, the cleavage property was lost in the ΔCAT mutant, reinforcing the importance of the catalytic activity of the proteinase for LDLR cleavage and consistent with our previous finding that the catalytically dead E240A mutant of MT1-MMP cannot cleave LDLR (18). Furthermore, deletion of the MT-loop essentially abolished MT1-MMP’s ability to cleave LDLR (lane 12 vs. 4). These findings indicate that the catalytic activity and the MT-loop are essential for MT1-MMP-promoted LDLR shedding.

### Mutational analysis of the MT-loop

**FIGURE 4:**
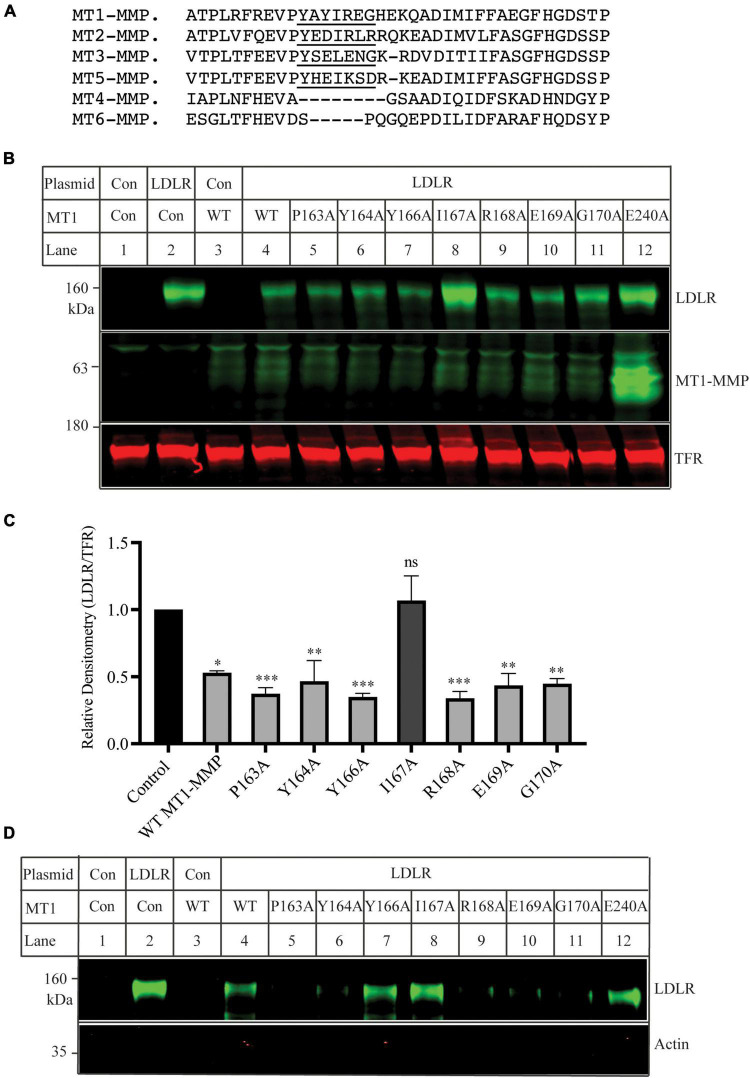
Mutational analysis of the MT-loop **(A)** Alignment of partial sequence of different MT-MMPs. The alignment was performed using Multiple Alignment (Fast Fourier Transform (MAFFT) FFT-NS-I, v7.429). MT1-MMP: NP_004986; MT2-MMP: NP_002419.1; MT3-MMP: NP_005932; MT5-MMP: NP_006681. MT4-MMP: NP_057239. MT6-MMP: NP_071913. The MT-loop was underlined. **(B**) Immunoblotting and **(C)** quantification. Plasmid DNA containing the wild-type LDLR was co-transfected with an equal amount of empty vector (Con), the wild-type, or mutant MT1-MMP into HEK293 cells using Lipofectamine 3000. 48 h later, whole cell lysate was prepared and applied to immunoblotting. LDLR and MT1-MMP were detected by a mouse monoclonal anti-LDLR antibody, HL-1, and a rabbit monoclonal anti-MT1-MMP antibody (abcam, ab51074), respectively. TFR, transferrin receptor (TFR), was recognized by a mouse anti-TFR monoclonal antibody. **(D)** Biotinylation. HEK293 cells were co-transfected with WT LDLR and WT or mutant MT1-MMP using Lipofectamine 3000. 48 h after, the cells were subjected to biotinylation and then immunoblotting using a mouse anti-LDLR (HL-1) and a mouse anti-actin antibody. Similar results were observed in three different experiments. Representative images were shown (B and D). Densitometry was determined by a Li-Cor Odyssey Infrared Imaging System. Relative densitometry was defined as the ratio of the densitometry of LDLR to that of TFR at the same condition. Values were mean ± S.D. of ≥3 experiments. The significant difference between two groups (wild type or mutant MT1-MMP vs. the Control) were determined *via* Student’s *t*-test. ns (no significance), *p* > 0.05, *p < 0.05, **p < 0.01, ***p < 0.001.

The MT-loop consists of seven amino acid residues and is present in all four membrane type MMPs (MT1-, MT2-, MT3-, and MT5-MMP), except the two glycosyl phosphatidylinositol-anchored membrane-associated MMPs (MT4- and MT6-MMP) (Figure 4A). Pro163 and Tyr164 in the MT-loop are completely conserved. We then performed alanine-scanning to determine the contribution of each of these amino acid residues to MT1-MMP’s action on LDLR. The mutant MT1-MMP and the wild-type LDLR were transfected into HEK293 cells using Lipofectamine™ 3000. A mouse monoclonal anti-LDLR (HL-1) detected the mature form of LDLR (Figures 4B,C). A rabbit anti-MT1-MMP monoclonal antibody was used to detect MT1-MMP. The antibody can recognize the full-length and self-cleaved MT1-MMP, consistent with a previous report (47). The levels of the catalytic dead mutation E240A were much higher than that of the wild-type and other mutant MT1-MMP since it loses the self-cleavage activity. We found that substitution of Ile167 with Ala (MT1-MMP^*I*167*A*^), like E240A, significantly impaired MT1-MMP’s ability to promote LDLR degradation, while mutation of other amino acid residues had no significant impact. Consistently, biotinylation experiments showed that I167A and E240A could not effectively reduce the levels of the mature form of LDLR when compared to the wild-type and other mutant MT1-MMP (Figure 4D). Taken together, our findings indicate the important role of Ile167 in MT1-MMP-promoted LDLR cleavage.

**FIGURE 5:**
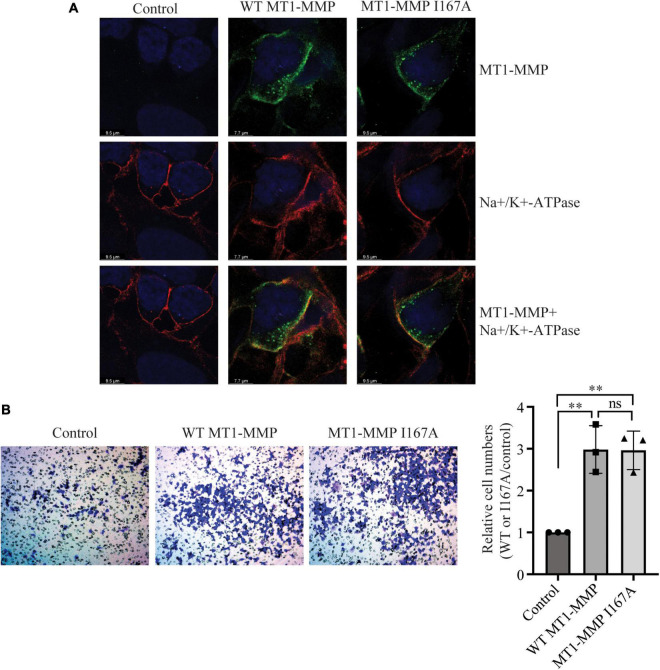
Effects of I167A on MT1-MMP trafficking and cell migration **(A)** Confocal microscopy. HEK293 cells transfected with empty plasmid (Control) or plasmid containing the HA-tagged wild-type or I167A mutant MT1-MMP were subjected to confocal microscopy. MT1-MMP was detected with a rabbit anti-HA polyclonal antibody (Proteintech, 51064-2-AP) and showed in green fluorescence (top and bottom panels). Na^+^/K^+^-ATPase was detected by a mouse monoclonal antibody and showed in red fluorescence (middle and bottom panels). Nuclei were visualized with DAPI (blue). An x-y optical section of the cells illustrates the distribution of the wild-type and mutant proteins (magnification: 325X). **(B)** Transwell assay. HEK293 cells were transfected with empty plasmid (Control) or plasmid containing the HA-tagged wild-type or I167A mutant MT1-MMP using Lipofectamine 3000 and then placed on a collagen type I-coated insert. 48 h after, cells were stained with crystal violet. Cells on the bottom of the insert were imaged, counted, and then divided by the image area. Relative cell numbers were the ratio of the cell numbers of cells transfected with the wild-type or I167A MT1-MMP to that of cells transfected with the empty vector (control), which was defined as 1. Representative images were shown. Values were mean ± S.D. of 3 experiments. The significant difference between two groups (wild type or mutant MT1-MMP vs. the Control) were determined *via* Student’s *t*-test. ns (no significance), *p* > 0.05, **p < 0.01.

We then performed confocal microscopy to assess whether mutation I167A affected the trafficking of MT1-MMP. As shown in Figure 5A, in cells transfected with empty vector (Control), MT1-MMP was undetectable by an anti-HA antibody that recognizes HA-tagged MT1-MMP. Both the wild-type MT1-MMP and MT1-MMP^*I*167*A*^ displayed a similar pattern, residing on the cell periphery and the intracellular space (green fluorescence in the top panel). We observed partial co-localization of the wild-type and mutant MT1-MMP with a plasma membrane marker, Na^+^/K^+^-ATPase (yellow fluorescence in the bottom panel). Next, we employed a transwell migration assay to assess the impact of mutation I167A on MT1-MMP-promoted cell migration. We observed that the relative cell numbers migrated through collagen type I-coated inserts were comparable in cells transfected with the wild-type MT1-MMP and MT1-MMP^*I*167*A*^; both significantly promoted cell migration compared to the control (Figure 5B). Therefore, substitution of Ile167 with Ala does not appear to affect MT1-MMP trafficking or its ability to promote cell migration.

### Detailed mutational analysis of Ile167

**FIGURE 6:**
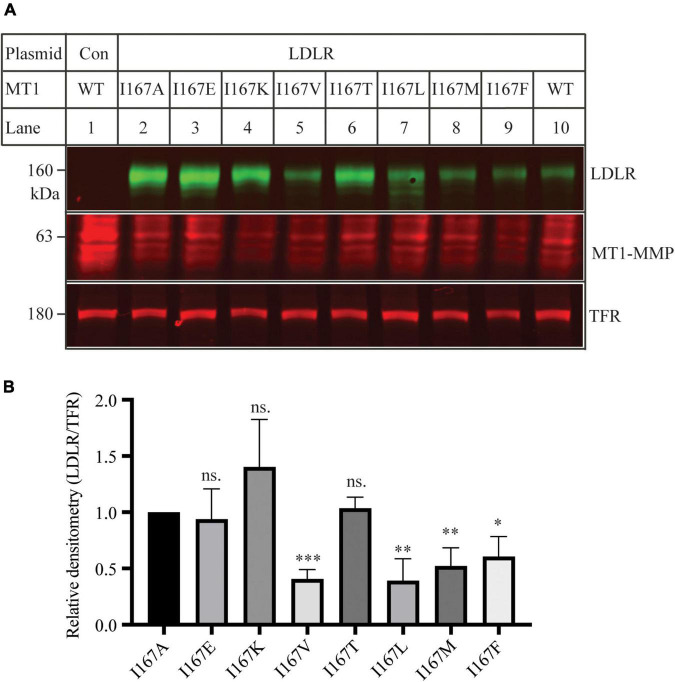
Mutational analysis of I167 **(A**) Immunoblotting and **(B)** quantification. Plasmid DNA containing wild-type LDLR was co-transfected with an equal amount of the wild-type or mutant MT1-MMP into HEK293 cells using Lipofectamine 3000. 48 h later, whole cell lysate was isolated and applied to immunoblotting with antibodies as described in the legend to Figure 5. TFR, transferrin receptor. Representative images were shown **(A)**. Densitometry was determined by a Li-Cor Odyssey Infrared Imaging System. Relative densitometry was defined as the ratio of the densitometry of LDLR to that of TFR at the same condition **(B)**. Values were mean ± S.D. of ≥3 experiments. The significant difference between two groups (mutations vs. I167A) were determined *via* Student’s *t*-test. ns (no significance), *p* > 0.05, *p < 0.05, **p < 0.01, ***p < 0.001.

Ile within the MT-loop of MT1-MMP is highly conserved in different species except for alligators and turtles. Instead of Ile, they have another hydrophobic residue, valine. Therefore, we investigated how specific the requirement was for Ile167 to contribute to MT1-MMP’s ability to promote LDLR cleavage. To assess whether another non-polar residue at position 167 could substitute for Ile, we replaced Ilel167 with a hydrophobic amino acid residue, including Leu, Val, Met, and Phe. We also mutated Ile167 to a polar residue, including Glu, Lys, and Thr, which has a negatively charged, a positively charged, and a polar side chain, respectively. Each of these mutant MT1-MMP was then co-expressed with the wild-type LDLR in HEK293 cells using Lipofectamine 3000. As shown in Figures 6A,B, like I167A, mutations I167E, I167K and I167T significantly impaired MT1-MMP-induced LDLR cleavage, whereas substitution of Ile with a hydrophobic residue (mutations I167V, I167L, I167M, and I167F) retained the ability to cleave LDLR. These findings indicate that a hydrophobic residue at position 167 within the MT-loop is required for MT1-MMP-promoted LDLR cleavage.

## Discussion

Our previous study demonstrates that hepatic MT1-MMP stimulates LDLR shedding, thereby increasing plasma LDL-C levels and the development of atherosclerosis (18). In addition, it has been reported that macrophage MT1-MMP degrades collagen in atherosclerotic plaques and consequently increases plaque vulnerability (48). On the other hand, MT1-MMP in vascular smooth muscle cells (VSMC) regulates extracellular matrix homeostasis and plays an important role in maintaining normal VSMC function (49). Deficiency of VSMC MT1-MMP increases the development of proliferative atherosclerotic lesions. Therefore, MT1-MMP appears to affect the development of atherosclerosis in a cell type-dependent manner, indicating the need for tissue/cell type-specific targeting of MT1-MMP to avoid undesired side effects. Specific targeting hepatocytes can be archived using N-acetylgalactosamine (GalNAc)-conjugation, such as GalNAc-siRNA or antisense oligonucleotides (ASOs) (50–52), which has been successfully used to silence hepatic PCSK9 expression in a clinical trial (51). GalNAc can mediate rapid hepatic uptake of conjugated targets *via* binding to the asialoglycoprotein receptor on hepatocytes (50). Thus, we can use GalNAc-siRNA or ASO to selectively target MT1-MMP in hepatocytes to inhibit LDLR shedding, thereby increasing clearance of plasma LDL-C and reducing the development of atherosclerosis.

The MMP family consists of 23 members, each with different physiological functions. To avoid potential off-target effects, a specific and selective MT1-MMP inhibitor is needed. This requires an in-depth understanding of the specificity of the protease cleavage site on their substrates. While no consensus cleavage site for MMPs has been identified, it is believed that MMPs preferentially cleave specific substrates at certain locations containing specific amino acid residues (53). Therefore, we first sought to identify the cleavage site of MT1-MMP on LDLR. However, our findings indicate that MT1-MMP does not need a specific cleavage site in the receptor.

It is of note that the catalytic core region is highly conserved among 23 MMP family members, which makes it very challenging to specifically inhibit the proteolytic activity of MT1-MMP by targeting this functional domain. Exosites have been shown to be a promising target for the development of specific MMP inhibitors. MT1-MMP contains several functional domains. The HPX domain contributes to the interaction of MT1-MMP with its substrates, such as CD44 and collagen (54–57). Targeting HPX with a small inhibitor can selectively inhibit MT1-MMP activity and suppress cancer cell growth (58). However, deletion of the HPX domain did not impair MT1-MMP-induced LDLR degradation. Conversely, the mutant appeared to promote LDLR degradation more strongly than the wild-type MT1-MMP (Figure 3). The exact underlying mechanism is unclear. The HPX domain is required for MT1-MMP binding to CD44 and collagen. These substrates may compete with LDLR for MT1-MMP binding. Therefore, it is possible that the deletion mutant can target LDLR more effectively than the wild-type MT1-MMP because it loses the ability to bind to other substrates.

The cytoplasmic tail of MT1-MMP plays an important role in endocytosis of MT1-MMP and the localization of MT1-MMP on the specific microdomains in the plasma membrane (19, 23–25). Deletion of the cytoplasmic tail did not affect the trafficking of MT1-MMP to the plasma membrane nor its ability to activate proMMP2, but the mutant proteins displayed a different distribution pattern on the cell surface and an impaired ability to mediate cell invasion compared to the wild-type protein (59, 60). However, removal of the C-terminal cytoplasmic tail of MT1-MMP did not significantly impair its ability to cleave LDLR. A similar phenotype was observed in the JD mutation that disrupts LDLR endocytosis. Therefore, MT1-MMP does not appear to induce LDLR cleavage during the endocytosis process.

**FIGURE 7:**
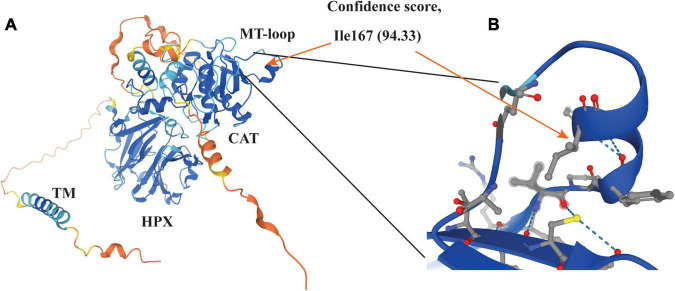
Structure of MT1-MMP **(A)** and enlarged view of the MT-loop **(B)** The structure was adopted from AlphaFold (https://alphafold.ebi.ac.uk/search/text/MT1-MMP) (68). The model confidence scores of Ile167 are very high (94.33). The side chain of Ile167 is pointed to the inside of the MT-loop (orange arrow in panel **B**).

Our previous study demonstrates that the catalytic activity of MT1-MMP is required for LDLR shedding (18). Consistently, we found that deletion of the catalytic domain of MT1-MMP virtually abolished its ability to cleave LDLR. Furthermore, our data indicate the requirement of a hydrophobic side chain at position 167 for MT1-MMP-induced LDLR cleavage. The MT-loop is flexible and only present in the four membrane type MMPs, but the conformation of the MT-loop is different in each membrane type MMP (61) and in MT1-MMP complexed with or without TIMPs (62–64). The crystallographic structure of the catalytic domain of MT1-MMP and the predicted structure of the full-length protein by AlphaFold reveal that the MT-loop protrudes from the main structure. Ile167 is situated in the middle of a small α-helix in the MT-loop with its hydrophobic side chain positioning inside the loop (Figure 7). How it contributes to MT1-MMP-induced LDLR cleavage is unclear. Weaver et al. reported that the MT-loop, together with the hemopexin domain, is required for the translocation of MT1-MMP from the apical to the basal membrane in polarized epithelial cells during tubulogenesis (65), but deletion of the entire MT-loop has no significant effect on the expression, trafficking, processing, or the proteolytic activity of MT1-MMP (61, 66). We also did not observe a significant difference in cell migration and MT1-MMP trafficking between the wild-type and I167A mutant MT1-MMP. These strongly suggest that I167A does not cause a major perturbation of the structure of the protein. However, we cannot exclude the possibility that I167A may result in a subtle structural change in the MT-loop. Nevertheless, the MT-loop represents a potential target for the development of selective MT1-MMP inhibitors due to its specificity and structurally easy accessibility. Indeed, an antibody specifically against the MT-loop has been developed and can block binding of pro-MMP2 to MT1-MMP and inhibit pro-MMP2 activation (67). It would be of interest to see if this antibody can block MT1-MMP-induced LDLR cleavage. In summary, although we did not find a specific cleavage site of MT1-MMP on LDLR, our findings on the role of the MT-loop in MT1-MMP’s action on LDLR provide critical information for the future design of highly sensitive and specific MT1-MMP inhibitors.

## Data availability statement

The original contributions presented in this study are included in the article/supplementary material, further inquiries can be directed to the corresponding author.

## Author contributions

D-WZ designed the experiments, analyzed the data, supervised and directed this project, and wrote the manuscript. MW, AA, H-MG, GG, ZZ, SJ, X-DX, and YS performed the experiments and collected and analyzed the data. G-QW and D-WZ provided the technical support and guidance and helpful discussions and comments. MW, AA, and D-WZ wrote the first draft of the manuscript. All authors contributed to the article and approved the submitted version.
